# 11. Vasculitis in a patient with intracranial myeloma

**DOI:** 10.1093/rap/rky033.003

**Published:** 2018-09-20

**Authors:** Shawki El-Ghazali, Kuljeet Bhamra, Ajmal Khan, Mitchell Burden, Vineela Nallapuneni, Ishita Patel

**Affiliations:** Rheumatology, Wexham Park Hospital, Slough, UNITED KINGDOM

**Introduction:** We present a case of a gentleman with a new diagnosis of intracranial plasma cell myeloma. PET scan as part of work-up identified the incidental presence of increased uptake primarily of the large vessel arterial system consistent with vasculitis. Review of the literature describes association of vasculitis and haematological malignancy (such as myelodysplastic syndrome and lymphoprolferative disorders), however the association with myeloma (and specifically large vessel vasculitis) appears to be less frequent with limited reported cases. We therefore wish to highlight this case for the interest of our rheumatological colleagues.

**Case description:** A 71 year old gentleman presented acutely with worsening vertigo symptoms and associated nausea. He had been experiencing such symptoms over the preceding two months, with his condition gradually progressing over that time. He had been experiencing intermittent right-sided occipital headaches, and following review from his GP during this period, he was started on gabapentin to manage a provisional diagnosis of occipital neuralgia. Apart from the above, he denied systemic features of weight loss, pyrexia, skin rash, myalgia or arthralgia. The patient had an extensive past medical history including monoclonal gammopathy of undetermined significance (IgG lambda paraprotein), IgG-mediated peripheral neuropathy, postural hypotension and previous diagnosis of polymyalgia rheumatica (in remission at time of presentation and not on therapy). Despite the above co-morbidities, prior to his recent symptoms, this gentleman was otherwise fit and well, and was only under observation in regards to his MGUS diagnosis via the local haematology team. On presentation, no obvious objective clinical abnormality could be identified including neurological deficit. GCS was 15/15. Routine blood tests were within normal range including inflammatory markers of CRP and ESR. In view of progressive vertigo symptoms, concerns of a possible intracranial lesion was considered, and as such arrangements were made for an urgent MRI head scan. This was reported to show an isointense, homogeneously enhancing destructive mass centred on the right occipital skull base, with a significant extraosseous component indenting the right cerebellar hemisphere with gross oedema. The lesion was approximately 6 cm in size. No hydrocephalus was present at time of the scan. Urgent neurosurgical opinion was advised and dexamethasone therapy (8 mg BD) was initiated. Neurosurgical review was arranged at the local centre, and biopsy of the lesion was performed. Histology demonstrated tissue diffusely infiltrated by plasma cells positive for CD138, CD79a and monotypic for lambda light chain - features were deemed consistent with plasma cell myeloma. Bone marrow trephine reported a small population of lambda light chain predominant plasma cells, confirmed by CD-138 potentially suggestive of myeloma. CT chest/abdomen/pelvis did not identify any obvious primary/secondary lesions. PET CT scan was arranged, which as well as the intrancranial lesion described, also reported generalised FDG uptake of the aorta, subclavian, axillary, common/external iliac and femoral arteries - findings were reported as consistent with large vessel vasculitis and were identified despite dexamethasone use. His case was referred to the haematology team for further management and decision has been made for radiotherapy treatment of the intracranial lesion. Incidental vasculitic changes described on PET scan have been attributed as secondary to his haematological condition, and arrangements are made for re-imaging following treatment.

**Discussion:** Associations between malignancy and vasculitis have been previously reported within the literature, with approximately 5-8% of vasculitis cases having associated malignancy. Reports suggest that haematological malignancies are more commonly associated than organ solid tumours, with sub-types such as MDS and lymphoma being most prominent. Myeloma related vasculitis appears to be less common, with limited described case reports. Various theories are made regarding the link of malignancy and vasculitis, with cause and affect between the two being unclear. For example, one potential association with myeloma is the development IL-6 genes resulting in leucocytoclastic vasculitis (LCV). LCV has been reported among the most common vasculitides associated with malignancy, however this is usually associated with small vessel cutaneous vasculitis. Indeed, large vessel vasculitis (as suggested by this case) related to myeloma appears to be extremely rare based on limited reports available. As per our described case, the patient did not appear to have findings suggestive of systemic vasculitis, and despite apparent extensive vascular involvement as per PET imaging, inflammatory markers remained normal throughout the presentation (even prior to dexamethasone usage) and appears to be an incidental finding from imaging. As mentioned, our patient is currently undergoing treatment for his primary intracranial lesion under the haematology team. Following therapy, it would be interesting to review whether apparent PET findings settle once the primary myeloma is managed.

**Key Learning Points:** The primary presentation for this patient focussed on his symptoms of persistent vertigo and associated nausea. Through extensive investigations involving multiple specialties, we were able to identify the causative lesion and histology suggestive of intracranial myeloma. Incidental finding of large vessel vasculitis on PET scan has highlighted a potential association between the two diagnoses. Myeloma and large vessel vasculitis appears to be rarely reported within the literature, and as such we feel this particular case may be of interest to the rheumatological community. Follow up on completion of myeloma treatment is planned to review possible resolution of PET findings.

**Disclosure: S. El-Ghazali:** None. **K. Bhamra:** None. **A. Khan:** None. **M. Burden:** None. **V. Nallapuneni:** None. **I. Patel:** None.

